# Can preoperative cervical spinal diffusion tensor imaging (DTI) indices predict surgical outcomes in patients with Hirayama disease? A retrospective cohort study

**DOI:** 10.3389/fneur.2022.982404

**Published:** 2022-09-30

**Authors:** Yuan Gao, Chi Sun, Shuyi Zhou, Xiaosheng Ma, Xinlei Xia, Feizhou Lu, Jun Zhang, Hongli Wang, Jianyuan Jiang

**Affiliations:** ^1^Department of Orthopedics, Huashan Hospital, Fudan University, Shanghai, China; ^2^Department of Radiology, Huashan Hospital, Fudan University, Shanghai, China

**Keywords:** Hirayama disease (HD), surgical outcome, diffusion tensor imaging (DTI), anterior cervical discectomy fusion (ACDF), Q-DASH score

## Abstract

**Objective:**

Anterior cervical discectomy and fusion (ACDF) surgery can effectively prevent disease progression in patients with Hirayama disease (HD) and diffusion tensor imaging (DTI) can quantitatively assess spinal cord function. In this study, we aimed to evaluate the relationship between preoperative spinal DTI indices and the clinical outcomes of patients with HD when treated by ACDF.

**Methods:**

We retrospectively analyzed 35 HD patients treated by ACDF. We collated a range of DTI indices, including fractional anisotropy (FA) and apparent diffusion coefficient (ADC) values, prior to surgery with patients in flexion and neutral positions. Patients were divided into improvement (Im) group and non-improvement (Nim) group according to the Odom score, and the difference in surgical outcomes between the two groups was confirmed by quick disabilities of the arm, shoulder and hand (Q-DASH) scores. The DTI indices in the two groups of patients were then compared. Receiver operating characteristic (ROC) curves and area under curve (AUC) were used to evaluate the predictive capability. The correlation between Q-DASH scores and DTI indices was also evaluated.

**Results:**

The FA values in the two groups of patients differed significantly in the cervical flexion position and the different segments were mainly located in the lower cervical spinal cord including the flexion C5/6 (Im group vs. Nim group: 0.501 ± 0.078 vs. 0.362 ± 0.087, *P* < 0.001) and C6/7 (Im group vs. Nim group: 0.455 ± 0.097 vs. 0.347 ± 0.102, *P* = 0.003) FA values, the mean FA value for C4/5-C6/7 (Im group vs. Nim group: 0.471 ± 0.067 vs. 0.372 ± 0.078, *P* < 0.001), mean FA value for C5/6-C6/7 (Im group vs. Nim group: 0.478 ± 0.076 vs. 0.354 ± 0.083, *P* < 0.001) and mean FA value for the two minimal segments (Im group vs. Nim group: 0.442 ± 0.078 vs. 0.341 ± 0.081, *P* = 0.001). The ADC values were similar to FA values. The ROC curve for DTI indices in the lower cervical spinal cord had an AUC > 0.7 including: flexion FA value and ADC value for C5/6 (0.877 and 0.931), flexion FA value and ADC value for C6/7 (0.778 and 0.761), flexion mean FA value and ADC value for C4/5-C6/7 (0.846 and 0.859), flexion mean FA value and ADC value for C5/6-C6/7 (0.861 and 0.905), flexion mean FA value and ADC value for the two minimal/maximal segments (0.815 and 0.892). DTI indices including FA value and ADC value were correlated with the preoperative score, final follow-up score and improvement percentage. Flexion ADC value was correlated with improvement score but flexion FA value was not correlated with improvement score.

**Conclusion:**

Preoperative DTI indices of the spinal cord, especially those of the lower cervical spinal cord with patients in the flexion position, can predict the clinical outcome of patients with HD post-surgery. In general, a larger FA value and a smaller ADC value indicate a better surgical outcome.

## Introduction

Hirayama disease (HD), also referred to as juvenile muscular atrophy of the distal upper extremity, was first reported by Keizo Hirayama in 1959 (1). This disease occurs mostly in adolescent males and is mainly characterized by atrophy of the intrinsic muscles of the hand and the forearm muscles on one or asymmetric sides, accompanied by mobility impairment (2). HD was previously considered a self-limiting disease that stops progressing 2–4 years after onset (3). Based on this opinion, conservative treatment such as collar therapy has become the primary treatment option for HD (4, 5). However, studies have shown that many patients with HD have persistent disease progression and conservative treatment is ineffective (6, 7). Therefore, some physicians have begun to use anterior cervical discectomy and fusion (ACDF) surgery to treat such patients because this procedure can limit excessive flexion of the cervical spine and reduce cervical instability (8–10).

Studies have shown that the cervical spinal cord diffusion tensor imaging (DTI) indices of patients with HD are significantly different from healthy volunteers and that the DTI indices of patients are also different when their neck is flexed and neutral; this may be related to their impaired spinal cord function (11). However, the existing literature has not evaluated the relationship between spinal cord DTI indices and surgical outcomes. In this study, we aimed to investigate whether preoperative spinal cord DTI indices could predict surgical outcomes in patients with HD. The results of this study might provide a reference for whether patients with HD need surgery.

## Materials and methods

### Patients

According to the clinician-led diagnosis and treatment guideline for HD (12), the diagnosis of HD should feature the following: (1) clinical manifestations such as unilateral intrinsic hand muscle atrophy, cold paralysis, extensor tremor or other symptoms; (2) cervical flexion magnetic resonance T2-weighted imaging (MR T2WI) in the sagittal plane showing crescent-shaped hyper-intensity behind the spinal cord, and (3) segmental and localized nerve damage of the anterior horn or anterior root in the lower cervical segments, as shown by electromyography (EMG).

**Figure 1:**
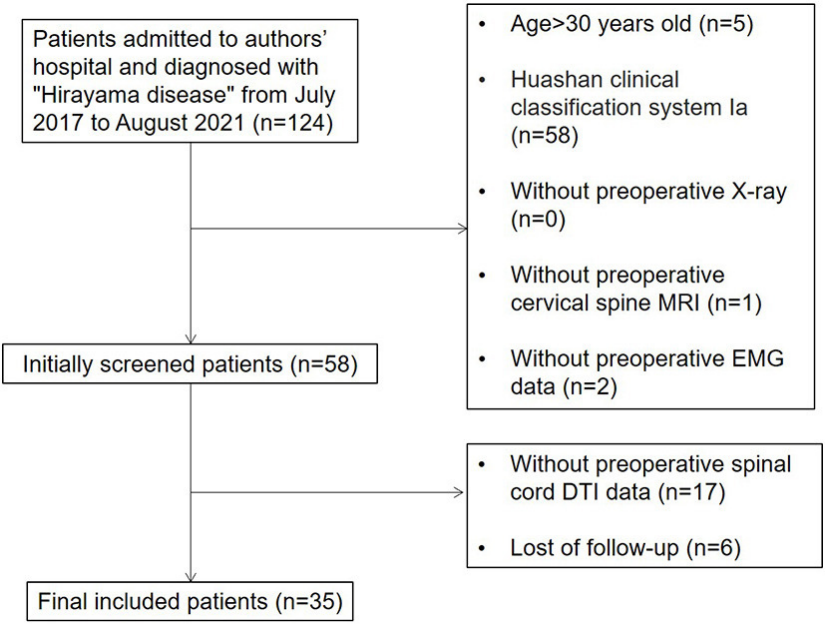
The inclusion process used in this study.

Based on the experience of treating almost 500 patients with HD, our institution established the Huashan clinical classification system for patients with HD and primarily verified the internal consistency of this classification system (13). According to this system, HD patients can be divided into types I, II and III. Patients with type I HD have typical symptoms such as unilateral muscular atrophy without pyramidal tract signs or sensory disturbances. If the symptoms do not progress within 6 months, the patient is classified as subtype Ia; however, if the patient progresses, then the classification is subtype Ib. Patients with type II HD have typical symptoms accompanied by sensory disturbances or pyramidal tract signs. Patients with type III HD have an atypical form of HD involving the proximal muscles of the upper limbs or bilateral symptoms. Therefore, we included patients who were clearly diagnosed with HD of type Ib or above in our institution from July 2017 to August 2021. Our inclusion process is shown in Figure 1.

The inclusion criteria were as follows: (1) the diagnosis was clear, with unilateral or bilateral distal upper extremity muscle weakness and atrophy; (2) flexion position T2WI MRI showed widening of the post-dural space, forming a crescent-shaped hyper-intensity zone in the sagittal plane with spinal cord thinning and atrophy in the horizontal plane; (3) EMG showed neurogenic lesions confined to the lower cervical spinal cord without abnormal peripheral nerve conduction velocities; (4) conservative treatment such as collar therapy had been ineffective for at least 6 months; (5) the disease progressed rapidly and seriously affected the patient's life since onset, and (6) patients were type Ib or above according to the Huashan clinical classification system.

The exclusion criteria were as follows: (1) the diagnosis was unclear; (2) symptoms improved after 6-month conservative treatment; (3) patients were unable to tolerate surgery due to other serious medical conditions, and (4) patients had contraindications to MRI such as claustrophobia or metal implants. Finally, a total of 35 cases were enrolled. Surgery was performed by doctors in the same specialty group.

### DTI protocol

We used a Siemens 3.0T MRI scanner (MAGNETOM Verio, Siemens, Germany) to acquire DTI images from the HD patients. First, we acquired neutral DTI images. Patients received the scan in a supine position with a fastened cervical coil. Then, we acquired flexion DTI images. After a 30-min neck flexion (patients were told to ensure that their chin was as close to the sternum as possible), patients received the scan while retaining neck flexion in the supine position. A single-shot echo-planar imaging (SS-EPI) sequence was used during the DTI scan. The imaging parameters were as follows: b value: 0 and 500 s/mm^2^; microwave pulse generation (MPG): 30 directions; repetition time (TR)/ echo time (TE): 2900/61 ms; sagittal section orientation; slice thickness/gap: 3/1 mm; field of view (FOV): 00 × 300 mm^2^; matrix: 128 × 128; actual voxel size: 2.0 × 2.0 × 2.0 mm^3^ and a scan time of 8 min 24 s. Sagittal anatomical T2W images were also acquired by a T2 turbo-spin-echo (TSE) sequence. The parameters were as follows: TR/TE: 2640/125.7 ms; FOV: 320 × 320 mm^2^; matrix: 128 × 128, and a slice thickness/gap of 3/1 mm.

### Post-processing of DTI data

**Figure 2:**
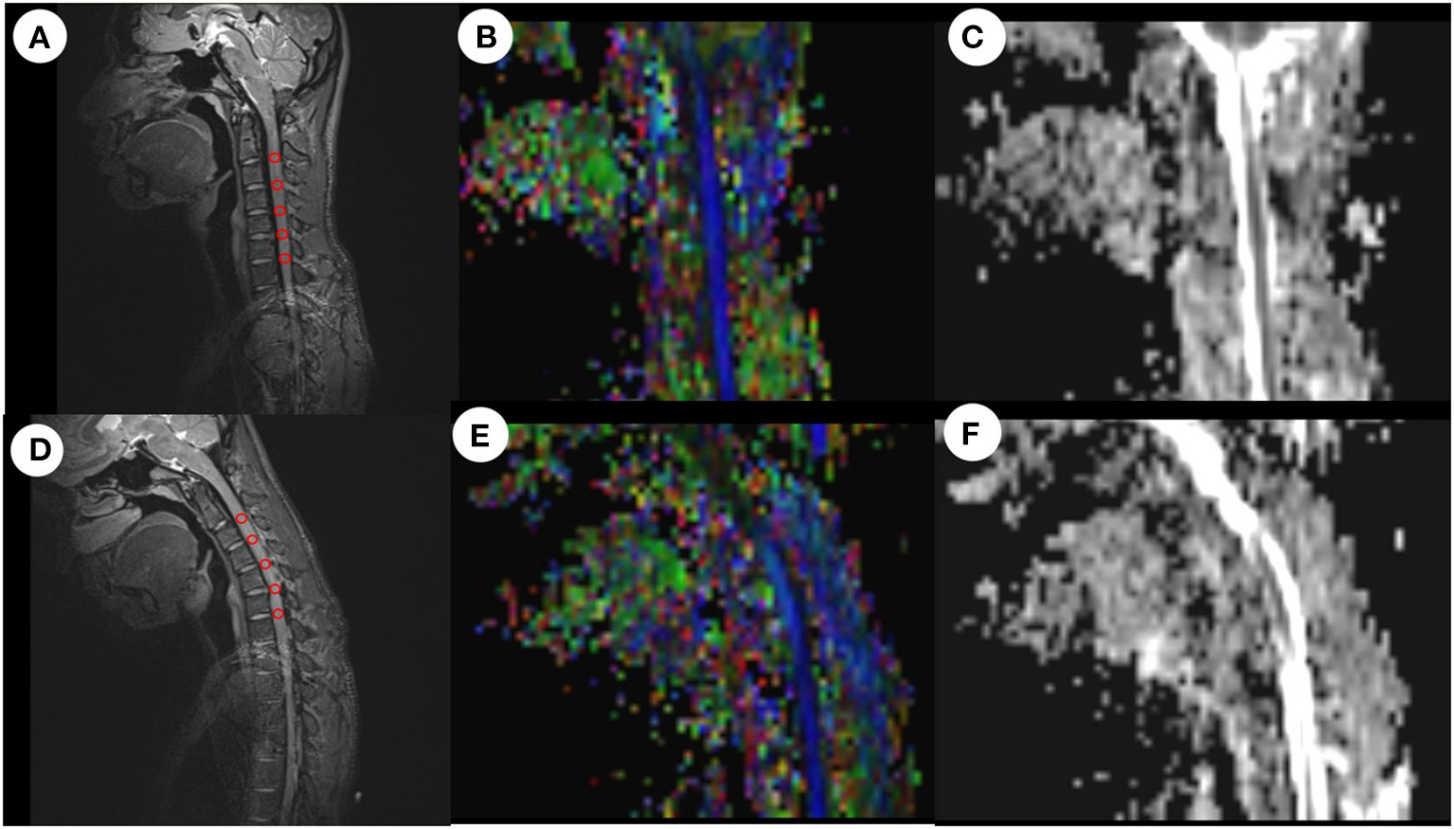
ROI-based measurement of FA values and ADC values in neutral and flexion positions. After drawing a ROI on the anatomical image, the system automatically calculated the mean FA value and mean ADC value for all voxels in the ROI. **(A)** Anatomical image in the neutral position. **(B)** FA image in the neutral position. **(C)** ADC image in the neutral position. **(D)** Anatomical image in the flexion position. **(E)** FA image in the flexion position. **(F)** ADC image in the flexion position. ROI, region of interest; FA, fractional anisotropy; ADC, apparent diffusion coefficient.

The acquired MRI images were post-processed in Siemens Healthcare AG (neuro 3D mode). We fused the anatomical T2WI images and DTI images with each other, found the mid-sagittal plane on the obtained fusion images, and drew a circular region of interest (ROI) in the spinal cord region on each segment of C2/3-C6/7 to determine a fractional anisotropy (FA) value and apparent diffusion coefficient (ADC) value for each ROI (Figure 2). It should be noted that each ROI could not exceed the spinal cord and each ROI needed to contain at least 3 voxels. We used the FA value and ADC value for each segment (C2/3-C6/7) to calculate the following 4 indices: (1) mean FA and ADC values for C2/3-C3/4; (2) mean FA and ADC values for C4/5-C6/7; (3) mean FA and ADC values for C5/6-C6/7, and (4) mean FA and ADC values for the two worst segments (FA values were two minimal segments and ADC values were two maximal segments). The measurements were determined independently by two doctors and the final result was the mean value calculated from the two doctors.

### Collection of preoperative EMG data

We collated EMG data for each patient prior to surgery. All neurophysiological measurements were performed by a Nihon Kohden MEB-940 EMG unit (Tokyo, Japan). The oscilloscope was scanned at a rate of 5 ms/cm with a magnification of 200–500 v/cm. The room temperature was controlled at 25°C and the skin temperature of the forearm was kept between 32°C and 34°C. To avoid the effect of inter-measurer variation, all EMG data was measured and collected by the same experienced physician. The median nerve was stimulated at the wrist and elbow. The ulnar nerve was stimulated at the wrist, above and below the elbow. During the motor nerve examination, the maximal compound muscle action potential (CMAP) values of the abductor pollicis brevis (APB) and first dorsal interosseous (FDI) were recorded during stimulation of the median and ulnar nerves. We also recorded the fibrillation and positive sharp wave of APB and FDI, presented as the number of plus signs.

### Surgery

All patients underwent ACDF surgery. After general anesthesia, the patient was placed in a supine position and a 3–4 cm transverse incision was made on the neck. After incising the subcutaneous tissue and muscles and pulling the trachea and esophagus to the other side, the cervical vertebra was fully exposed. The use of a guide pin and intraoperative fluoroscopy was used to help determine the operative segments. After removing the intervertebral disc and the cartilage endplate of the diseased segments, we took autologous iliac bone from the patient's anterior superior iliac crest and inserted this into the intervertebral cage. Then, we implanted the cage into the space where the intervertebral disc had been excavated. Finally, we installed a titanium plate and fixed it with screws. The posterior longitudinal ligament was fully preserved during the operation. The choice of surgical segment was determined by combining the patient's clinical symptoms, preoperative imaging examination and preoperative EMG examination. All patients included in this study underwent anterior fusion and internal fixation of the lower cervical spine (Table 1).

**Table 1:** Baseline characteristics, follow-up time and surgical segments of the two groups of patients.

	**Im group**	**Nim group**	***P*-value**
Number of cases	18	17	/
Age (years)	18.44 ± 1.97	20.41 ± 3.54	0.047^*^
Height (cm)	171.56 ± 7.42	173.94 ± 5.04	0.277
Weight (kg)	61.00 ± 11.14	66.06 ± 9.20	0.154
Gender (male%)	88.89%	100%	0.486
Age of disease onset (years)	15.83 ± 1.72	16.88 ± 3.10	0.841
Follow-up time (months)	38.22 ± 13.17	28.22 ± 15.56	0.059
Surgical segments	/	/	/
Cases of C4/5-C5/6	10	8	/
Cases of C5/6-C6/7	7	8	/
Cases of C4/5-C6/7	1	1	/

Gender is presented as the percentage of males; cases are presented as frequency; other values are presented as mean ± standard deviation. Im, improvement; Nim, non-improvement.

* *P* < 0.05.

### Follow-up and evaluation of surgical outcomes

All patients were followed up and we ensured they had at least 6 months postoperative recovery. The main items recorded during follow-up were quick disabilities of the arm, shoulder and hand (Q-DASH) score and the Odom score. The patients also completed the Q-DASH scoring questionnaire before surgery. The Odom score is a questionnaire that judges the surgical outcomes based on the patient's own subjective feelings. The specific items are as follows: grade 4 - no symptoms related to cervical disease and able to perform daily activities without limitations; grade 3 - moderate symptoms related to cervical disease and able to perform daily activities without significant limitations; grade 2 - slight improvement in symptoms related to cervical disease and significant limitations in daily activities and grade 1 - non-improvement in, or aggravation of symptoms related to cervical disease and unable to perform daily activities. According to the score, grade 4 and grade 3 were included in the improvement (Im) group while grade 2 and grade 1 were included in the non-improvement (Nim) group. Considering the subjectivity of the Odom score, we also compared the patients' preoperative Q-DASH scores and their final follow-up scores and calculated their improvement scores and improvement percentages to confirm the difference in surgical outcomes between the two groups.

### Statistical analysis

We used IBM SPSS Statistics for Windows, version 23.0 (IBM Corp., Armonk, N.Y., USA) for statistical analysis. Gender was evaluated by Fisher's exact test. All other data were evaluated by the *t*-test. For data that did not meet the criteria for the *t*-test, the Wilcoxon rank-sum test was applied. We also drew a receiver operating characteristic (ROC) curve using the flexion DTI indices that were statistically different between the two groups of patients, and calculated the cut-off value, sensitivity and specificity. Finally, we performed Spearman's correlation analysis between the flexion DTI indices and the Q-DASH score. *P* < 0.05 was considered statistically significant.

## Results

### Patient follow-up and the evaluation of surgical outcomes

According to the inclusion and exclusion criteria, a total of 35 patients were finally included. According to the Odom score, 35 patients were scored as follows: grade 4 (5 cases), grade 3 (13 cases), grade 2 (15 cases) and grade 1 (2 cases); this allowed us to generate two study groups (Im group and Nim group). The baseline characteristics, follow-up time and surgical segment information of the two groups of patients are shown in Table 1. The comparison of Q-DASH scores between the two groups is shown in Table 2. Although the preoperative score was significant different, the difference in improvement score and in the improvement percentage showed that the two groups of patients experienced different surgical outcomes.

**Table 2:** Comparison of Q-DASH scores between the two groups of patients.

	**Im group**	**Nim group**	***P*-value**
Preoperative score	41.67 ± 10.29	57.79 ± 6.78	< 0.001^***^
Final follow-up score	31.25 ± 8.76	55.73 ± 6.66	< 0.001^***^
Improvement score	10.42 ± 4.72	2.06 ± 2.02	< 0.001^***^
Improvement percentage	25.19 ± 8.72%	3.52 ± 3.61%	< 0.001^***^

Values are presented as mean ± standard deviation. Q-DASH, quick-disabilities of the arm, shoulder and hand; Im, improvement; Nim, non-improvement. Improvement score = final follow-up score - preoperative score; improvement percentage = improvement score / preoperative score.

*** *P* < 0.001.

### Comparison of preoperative EMG data between the two groups of patients

A comparison of preoperative EMG data between the two groups of patients is shown in Table 3. We compared four indices, including the CMAP amplitude of the ulnar nerve, the CMAP amplitude of the median nerve, fibrillation and positive sharp waves of FDI and fibrillation and positive sharp waves of ABD. We found that only the CMAP amplitude of the ulnar nerve differed significantly between the two groups (Im group vs. Nim group: 5.92 ± 4.59 vs. 3.29 ± 2.34, *P* = 0.041).

**Table 3:** Comparison of preoperative EMG data between the two groups of patients.

	**Im group**	**Nim group**	***P*-value**
CMAP (ulnar nerve)	5.92 ± 4.59	3.29 ± 2.34	0.041^*^
CMAP (median nerve)	10.63 ± 5.01	7.75 ± 4.80	0.092
Amount of fibrillation and positive sharp wave (FDI)	1.78 ± 1.17	1.53 ± 1.24	0.526
Amount of fibrillation and positive sharp wave (APB)	1.39 ± 1.09	1.41 ± 1.18	0.953

Values are presented as mean ± standard deviation. Im, improvement; Nim, non-improvement; EMG, electromyography; CMAP, maximal compound muscle action potential; FDI, first dorsal interosseous; APB, abductor pollicis brevis.

* *P* < 0.05.

### Comparison of preoperative DTI indices between the two groups of patients

**Figure 3:**
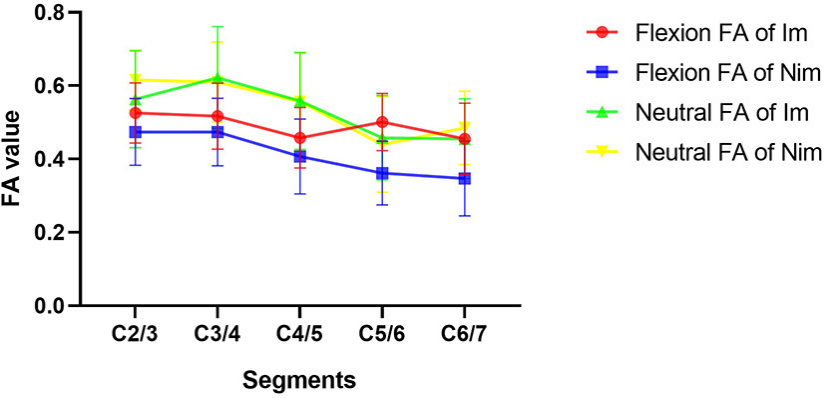
Comparison of FA values between the two groups of patients. The figure shows the trends in variation of FA values from C2/3 to C6/7. Im, improvement group; Nim, non-improvement group; FA, fractional anisotropy.

**Figure 4:**
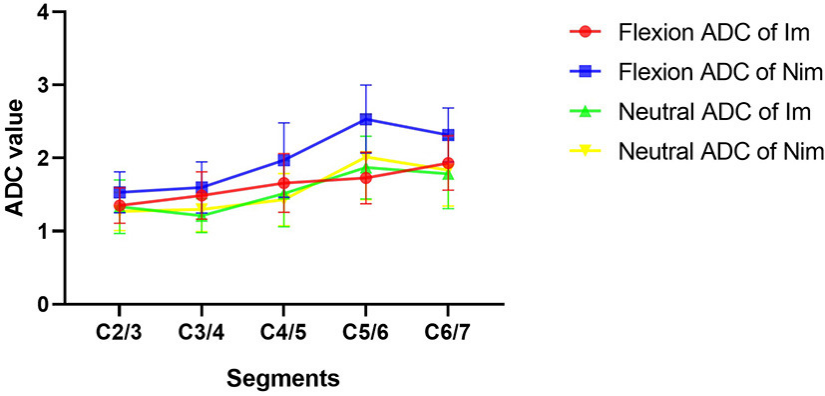
Comparison of ADC values between the two groups of patients. The figure shows the trends in variation of ADC values from C2/3 to C6/7. Im, improvement group; Nim, non-improvement group; ADC, apparent diffusion coefficient.

Comparison of flexion and neutral FA values between the two groups of patients is shown in Table 4. The flexion C5/6 (Im group *vs*. Nim group: 0.501 ± 0.078 vs. 0.362 ± 0.087, *P* < 0.001) and C6/7 (Im group *vs*. Nim group: 0.455 ± 0.097 vs. 0.347 ± 0.102, *P* = 0.003) FA values were significantly different between the two groups. The lower cervical indices, including the mean FA value for C4/5-C6/7 (Im group *vs*. Nim group: 0.471 ± 0.067 vs. 0.372 ± 0.078, *P* < 0.001), mean FA value for C5/6-C6/7 (Im group vs. Nim group: 0.478 ± 0.076 vs. 0.354 ± 0.083, *P* < 0.001) and mean FA value for the two minimal segments (Im group vs. Nim group: 0.442 ± 0.078 vs. 0.341 ± 0.081, *P* = 0.001) were also significantly different. The neutral FA values were not significantly different. The ADC values were similar to FA values (Table 5) and there were also significant differences in flexion C2/3 (Im group vs. Nim group: 1.351 ± 0.241 vs. 1.531 ± 0.279, *P* = 0.048) and in flexion C4/5 (Im group vs. Nim group: 1.659 ± 0.401 vs. 1.973 ± 0.507, *P* = 0.050). The trend in variation for the C2/3-C6/7 FA value is shown in Figure 3 while the trend in variation for the C2/3-C6/7 ADC value is shown in Figure 4.

**Table 4:** Comparison of flexion and neutral FA values between the two groups of patients.

	**Flexion FA value**			**Neutral FA value**		
	**Im group**	**Nim group**	***P*-value**	**Im group**	**Nim group**	***P*-value**
C2/3	0.526 ± 0.082	0.474 ± 0.091	0.082	0.563 ± 0.132	0.616 ± 0.082	0.163
C3/4	0.517 ± 0.090	0.474 ± 0.092	0.174	0.622 ± 0.139	0.610 ± 0.109	0.792
C4/5	0.458 ± 0.082	0.407 ± 0.102	0.112	0.558 ± 0.132	0.556 ± 0.133	0.957
C5/6	0.501 ± 0.078	0.362 ± 0.087	< 0.001^***^	0.458 ± 0.115	0.440 ± 0.130	0.672
C6/7	0.455 ± 0.097	0.347 ± 0.102	0.003^**^	0.455 ± 0.109	0.485 ± 0.100	0.389
Mean of C2/3-C3/4	0.521 ± 0.075	0.474 ± 0.084	0.085	0.592 ± 0.120	0.613 ± 0.073	0.537
Mean of C4/5-C6/7	0.471 ± 0.067	0.372 ± 0.078	< 0.001^***^	0.490 ± 0.086	0.494 ± 0.095	0.907
Mean of C5/6-C6/7	0.478 ± 0.076	0.354 ± 0.083	< 0.001^***^	0.456 ± 0.092	0.463 ± 0.099	0.838
Mean of two minimal segments	0.442 ± 0.078	0.341 ± 0.081	0.001^**^	0.438 ± 0.084	0.445 ± 0.106	0.826

Values are presented as mean ± standard deviation. Im, improvement; Nim, Non-improvement; FA, fractional anisotropy.

** *P* < 0.01,

*** *P* < 0.001.

**Table 5:** Comparison of flexion and neutral ADC values between the two groups of patients.

	**Flexion ADC value**			**Neutral ADC value**		
	**Im group**	**Nim group**	***P*-value**	**Im group**	**Nim group**	***P*-value**
C2/3	1.351 ± 0.241	1.531 ± 0.279	0.048^*^	1.336 ± 0.367	1.271 ± 0.261	0.552
C3/4	1.488 ± 0.322	1.598 ± 0.351	0.342	1.213 ± 0.231	1.298 ± 0.304	0.360
C4/5	1.659 ± 0.401	1.973 ± 0.507	0.050^*^	1.514 ± 0.451	1.431 ± 0.357	0.551
C5/6	1.728 ± 0.353	2.533 ± 0.466	< 0.001^***^	1.870 ± 0.429	2.014 ± 0.583	0.407
C6/7	1.933 ± 0.372	2.316 ± 0.368	0.004^**^	1.786 ± 0.477	1.836 ± 0.492	0.766
Mean of C2/3-C3/4	1.419 ± 0.244	1.564 ± 0.294	0.121	1.275 ± 0.243	1.285 ± 0.239	0.906
Mean of C4/5-C6/7	1.773 ± 0.305	2.274 ± 0.342	< 0.001^***^	1.723 ± 0.305	1.760 ± 0.380	0.753
Mean of C5/6-C6/7	1.831 ± 0.314	2.424 ± 0.335	< 0.001^***^	1.828 ± 0.366	1.925 ± 0.491	0.511
Mean of two maximal segments	1.900 ± 0.309	2.484 ± 0.378	< 0.001^***^	1.932 ± 0.315	1.961 ± 0.465	0.829

Values are presented as mean ± standard deviation. Im, improvement; Nim, non-improvement; ADC, apparent diffusion coefficient.

* *P* < 0.05,

** *P* < 0.01,

*** *P* < 0.001.

### ROC curve analysis and prediction threshold

**Figure 5:**
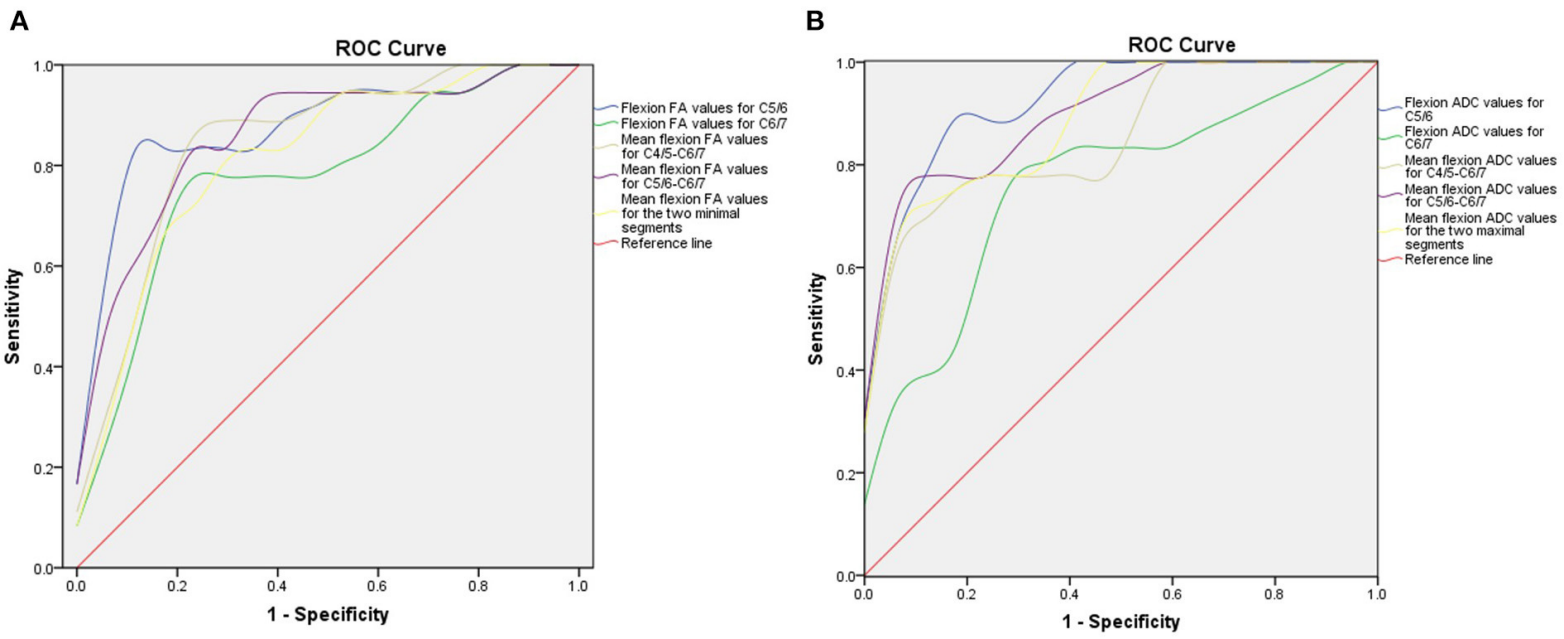
ROC curve for preoperative DTI parameters. **(A)** ROC curve of preoperative FA values in the flexion position. **(B)** ROC curve of preoperative ADC value in the flexion position. ROC, receiver operating characteristic; DTI, diffusion tensor imaging; FA, fractional anisotropy; ADC, apparent diffusion coefficient.

Next, we generated ROC curves with the flexion DTI indices (Figure 5). ROC curve analysis showed that the following indices achieved an AUC > 0.7: flexion FA value and ADC value for C5/6 (0.877 and 0.931), flexion FA value and ADC value for C6/7 (0.778 and 0.761), flexion mean FA value and ADC value for C4/5-C6/7 (0.846 and 0.859), flexion mean FA value and ADC value for C5/6-C6/7 (0.861 and 0.905), flexion mean FA value and ADC value for the two minimal/maximal segments (0.815 and 0.892). The cut-off value, sensitivity and specificity of these indices are shown in Tables 6, 7.

**Table 6:** Parameters derived from ROC analysis according to flexion FA value.

	**C5/6**	**C6/7**	**Mean of C4/5-C6/7**	**Mean of C5/6-C6/7**	**Mean of two minimal segments**
AUC	0.877	0.778	0.846	0.861	0.815
95% CI	0.755–1.000	0.619–0.936	0.712–0.980	0.735–0.987	0.671–0.959
YI	0.774	0.602	0.657	0.657	0.546
Cut-off value	0.463	0.401	0.414	0.420	0.396
Sensitivity	0.833	0.778	0.833	0.833	0.722
Specificity	0.941	0.824	0.824	0.824	0.824

ROC, receiver operating characteristic; FA, fractional anisotropy; AUC, area under curve; CI, confidence interval; YI, Youden index.

**Table 7:** Parameters derived from ROC curve analysis according to flexion ADC value.

	**C5/6**	**C6/7**	**Mean of C4/5-C6/7**	**Mean of C5/6-C6/7**	**Mean of two maximal segments**
AUC	0.931	0.761	0.859	0.905	0.892
95% CI	0.853–1.000	0.599–0.924	0.737–0.982	0.809–1.000	0.789–0.995
YI	0.771	0.543	0.608	0.719	0.663
Cut-off value	2.072	2.056	1.802	2.009	2.009
Sensitivity	0.889	0.778	0.667	0.778	0.722
Specificity	0.882	0.765	0.941	0.941	0.941

ROC, receiver operating characteristic; ADC, apparent diffusion coefficient; AUC, area under curve; CI, confidence interval; YI, Youden index.

### Correlation analysis between DTI indices and Q-DASH scores

**Figure 6:**
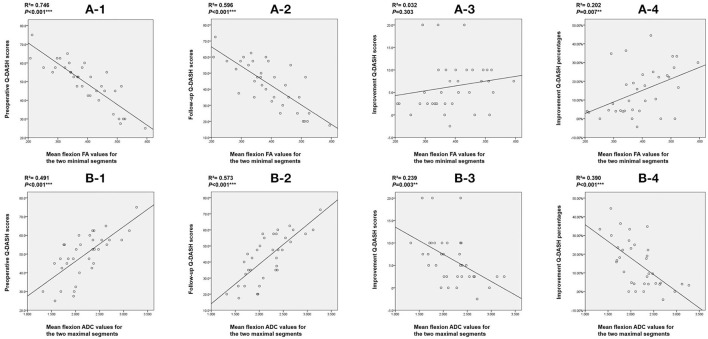
Correlation between DTI indices and Q-DASH scores. **(A)** Correlation between mean flexion FA value for the two minimal segments and (1) preoperative Q-DASH score; (2) final follow-up Q-DASH score; (3) improvement Q-DASH score; (4) improvement Q-DASH percentage. **(B)** Correlation between mean flexion ADC value for the two maximal segments and (1) preoperative Q-DASH score; (2) final follow-up Q-DASH score; (3) improvement Q-DASH score, and (4) improvement Q-DASH percentage. DTI, diffusion tensor imaging; Q-DASH, quick- disabilities of the arm, shoulder and hand; FA, fractional anisotropy; ADC, apparent diffusion coefficient.

Finally, we performed Spearman's correlation analysis using preoperative Q-DASH scores, follow-up Q-DASH scores, improvement scores and improvement percentages with mean flexion FA values for the two minimal segments and mean flexion ADC values for the two maximal segments. We found that DTI indices including FA value and ADC value were correlated with the preoperative score, final follow-up score and improvement percentage. Flexion ADC value was correlated with improvement score but flexion FA value was not correlated with improvement score (Figure 6).

## Discussion

Although the pathogenesis of HD is not fully understood, two main hypotheses have been proposed: the dynamic cervical flexion compression hypothesis and the growth and development hypothesis (14, 15). Both of these theories consider that cervical flexion is an important pathogenic factor for HD. Based on these theories, many physicians now use cervical collar therapy to treat HD (16, 17). However, the outcomes of cervical collar therapy are significantly influenced by patient compliance (18). Furthermore, reports have shown that some patients experience the continuous progression of symptoms for up to 10 years after the onset of this disease (6). Also, a small number of patients experience rapid progression within months or years of the initial symptoms (7). For such patients, many physicians have begun to perform ACDF surgery and have demonstrated that patients can benefit from this form of surgery (8–10, 19). In addition, a large number of studies, including guidelines established by clinical multidisciplinary teams, have reported that surgical treatment can effectively limit abnormal flexion of the cervical spine and expand the volume of the dural sac (12, 20). Based on these studies, many HD patients who have failed conservative treatment have subsequently benefited from surgery. Many physicians have begun to follow up HD patients who underwent ACDF surgery in the early years (21–24) and have found that although most patients can benefit from ACDF surgery, the surgical outcomes tended to differ among these patients.

The main symptom of patients with HD is atrophy of the hand muscles. Based on this, some physicians determine surgical outcomes by following up their patients by EMG data (24). However, EMG data can be affected by temperature, humidity and operator experience (25). Therefore, EMG data can only be used as a semi-quantitative assessment. On the other hand, the surgical outcomes of ACDF in HD patients have been proven to be correlated with spinal cord atrophy indices (26, 27) and cervical sagittal balance indices (28) although these studies only used non-quantitative measurements and focused on the morphology of the spinal cord or the stability of the cervical spine. The direct lesion in HD patients involves their spinal cord and the functional impairment of the spinal cord is the earliest and most sensitive symptom. Therefore, preoperative quantitative assessment of the patient's spinal cord function is the best way to predict surgical outcome.

DTI can be used to non-invasively examine neurological function and has become an established form of functional brain imaging. In recent years, many physicians have used DTI to study the spinal cord. For example, Yoo et al. compared spinal cord DTI indices in patients with cervical spondylotic myelopathy and healthy volunteers (29). These authors found that DTI provided a better relationship between spinal cord status and clinical manifestations than conventional T2-weighted MRI. In another study, Gao et al. found that FA value and fiber tract tracking of DTI were more sensitive to spinal cord lesions than T2WI and was positively correlated with clinical symptoms (30). Sun et al. utilized DTI to prove the hypothesis of dynamic cervical flexion compression and found that DTI spinal cord imaging can non-invasively and objectively reflect the functional state of the spinal cord (11). However, no studies have focused on the evaluation of surgical outcomes in HD patients using preoperative DTI indices. Thus, in our study, we aimed to compare the DTI indices of HD patients with different surgical outcomes and to determine whether DTI indices can be used as predictors to evaluate the surgical outcomes of HD patients.

To judge the surgical outcomes of patients, it is necessary to evaluate the upper extremity motor function of patients after ACDF. Previous studies used the Odom score to evaluate the surgical satisfaction of cervical spondylotic myelopathy (31). Over recent years, many physicians have applied this score to the evaluation of postoperative efficacy in patients with HD (22, 26, 28). Thus, in our present study, patients were divided into two groups (Im group and Nim group) according to the easy-to-use Odom score. Considering the subjectivity of the Odom score, we also collated the preoperative Q-DASH scores and final follow-up scores and calculated improvement scores and improvement percentages. According to the results, we found that although the preoperative Q-DASH scores in the two groups of patients were significantly different, thus indicating significant differences in preoperative upper extremity motor function, there were also differences in final follow-up scores, improvement scores and improvement percentages. These data indicated that patients achieved different surgical outcomes and experiences different postoperative upper extremity functions. Therefore, the Odom score was effective.

According to the comparison results of DTI indices between the two groups of patients, we found that the flexion FA value and ADC value for C5/C6, flexion FA value and ADC value for C6/7, mean flexion FA value and ADC value for C4/5-C6/7, mean flexion FA value and ADC value for C5/6-C6/7 and mean flexion FA value and ADC value for the worst two segments showed statistically significant differences. We believe that DTI indices represent the functional status of a patient's spinal cord. These differences indicate that the preoperative spinal cord function impairment status of the two groups of HD patients was different. Larger FA and smaller ADC values suggest better spinal cord function and also predict better surgical outcomes. ROC curve analysis further showed that most of the lower cervical spinal cord DTI indices achieved AUCs greater than 0.7. The AUC for the flexion ADC value for the C5/6 segment was > 0.9 with a cut-off value of 2.072, a sensitivity of 88.9% and a specificity of 88.2%. Furthermore, correlation analysis showed that the DTI indices for the lower cervical spinal cord exhibited a strong correlation with the preoperative Q-DASH score (R^2^ = 0.746; R^2^ = 0.491) and the follow-up Q-DASH score (R^2^ = 0.596; R^2^ = 0.573), thus indicating that preoperative spinal cord function was related to upper limb function. There was also a correlation between DTI indices and improvement percentage (R^2^ = 0.202; R^2^ = 0.390), thus indicating that preoperative spinal cord function was related to surgical outcomes.

Our study also has some limitations that need to be considered. Firstly, we used ROI-based DTI analysis to collect spinal cord DTI indices. This method cannot distinguish between gray and white matter. Future studies may require more advanced methods to study the lesion site. Secondly, we used the Odom score to evaluate surgical outcomes. Although we confirmed the results with preoperative and postoperative Q-DASH scores, a more objective approach still needs to be identified. Finally, we used univariate analysis and did not consider the influence of other factors such as cervical sagittal balance and spinal cord morphology.

## Conclusion

Preoperative DTI indices of the spinal cord, especially the indices of the lower cervical spinal cord when patients are in the flexion position, can reflect the functional status of the spinal cord in HD patients and can thus allow for better evaluation of the surgical outcomes of patients. In general, a larger FA value and a smaller ADC value indicate a better neurological function and a better surgical outcome.

## Data availability statement

The raw data supporting the conclusions of this article will be made available by the authors, without undue reservation.

## Ethics statement

The studies involving human participants were reviewed and approved by the Ethics Committee of Huashan Hospital Affiliated to Fudan University. Written informed consent to participate in this study was provided by the participants' legal guardian/next of kin. Written informed consent was obtained from the individual(s), and minor(s)' legal guardian/next of kin, for the publication of any potentially identifiable images or data included in this article.

## Author contributions

JZ and JJ contributed to the design of the study. YG contributed to the data collection, analysis, and manuscript writing. CS contributed to the data analysis and manuscript writing. SZ contributed to the data collection and analysis. HW contributed the funding supporting. JZ, JJ, XM, XX, and FL contributed to the final review of the manuscript. All authors contributed to the article and approved the submitted version.

## Funding

This study was funded by Clinical Technology Innovation Project of Shanghai Hospital Development Center (HW, No. SHDC12019X26), the Clinical Research Plan of Shanghai Hospital Development Center (HW, No. SHDC2020CR4030), National Natural Science Foundation of China (JJ, No. 82072488), AO Spine National Research Grant 2022 (CS, No. AOSCNR202219), and AO Spine National Research Grant 2020 (HW, No. AOSCN(R)2020-09).

## Conflict of interest

The authors declare that the research was conducted in the absence of any commercial or financial relationships that could be construed as a potential conflict of interest.

## Publisher's note

All claims expressed in this article are solely those of the authors and do not necessarily represent those of their affiliated organizations, or those of the publisher, the editors and the reviewers. Any product that may be evaluated in this article, or claim that may be made by its manufacturer, is not guaranteed or endorsed by the publisher.
